# Methodological quality and transparency of clinical practice guidelines for the pharmacological treatment of non-communicable diseases using the AGREE II instrument: a systematic review protocol

**DOI:** 10.1186/s13643-017-0621-5

**Published:** 2017-11-02

**Authors:** Caroline de Godoi Rezende Costa Molino, Eliane Ribeiro, Nicolina Silvana Romano-Lieber, Airton Tetelbom Stein, Daniela Oliveira de Melo

**Affiliations:** 1 Department of Pharmacy, Faculty of Pharmaceutical Sciences, University of São Paulo, Av. Professor Lineu Prestes, 580, São Paulo, SP 05508-000 Brazil; 2 Department of Politics, Management and Health, School of Public Health, University of Sao Paulo, Av. Dr. Arnaldo, 715, São Paulo, SP 01246-904 Brazil; 3 Department of Public Health, Federal University of Health Sciences of Porto Alegre, Rua Sarmento Leite, 245 -, Porto Alegre, RS 90050-170 Brazil; 4 Department of Biological Sciences, Institute of Environmental Sciences, Chemical and Pharmaceutical, Federal University of São Paulo (UNIFESP), Avenida Conceição, 329, Centro, Diadema, SP 09920-000 Brazil

**Keywords:** Practice guideline, AGREE instrument, Review, Primary health care, Chronic disease, Clinical protocols

## Abstract

**Background:**

Non-communicable diseases (NCDs) are the leading cause of death worldwide. Clinical practice guidelines (CPGs) constitute an important tool for the promotion of evidence-based health, which may improve healthcare outcomes for individuals with NCDs. Studies have shown that many CPGs have poor or moderate quality. Therefore, the aim of the proposed study is to systematically identify and appraise CPGs for pharmacological treatment of the most prevalent NCDs in primary care.

**Methods:**

A comprehensive literature search will be conducted in the following databases: MEDLINE, Embase and Cochrane Library. Twelve databases specific to CPGs will also be searched. Three appraisers will assess the quality of the CPGs using the Appraisal of Guidelines Research and Evaluation (AGREE) Instrument, version II. The AGREE II results will be checked for discrepancies. Differences between scores equal than or greater to 2 will be considered discrepant and the appraisers will decide the final score by consensus. If no consensus is reached, a fourth appraiser will decide the score. According to the AGREE II User’s Manual, the six domains of the instrument are independent. Thus, each domain score will be calculated by the sum of the individual item scores and scaling the total as a percentage of the maximum possible score for the domain.

**Discussion:**

The AGREE II instrument will be applied to evaluate the quality of CPGs and contribute to enhance the discussion and development of guidelines of high quality. The findings will be submitted for publication in high-impact, peer-reviewed scientific journals and will also be disseminated at international conferences.

**Systematic review registration:**

PROSPERO CRD42016043364

**Electronic supplementary material:**

The online version of this article (10.1186/s13643-017-0621-5) contains supplementary material, which is available to authorized users.

## Background

Non-communicable diseases (NCDs) are the major cause of death worldwide, accounting for 70% of deaths in 2015 [1]. The most prevalent NCDs are cardiovascular disease, cancer, respiratory disease, type 2 diabetes mellitus and mental illness [1–5]. The burden is higher in low- and middle-income countries, since the impact on the expenditures of households is higher and nearly three quarters of NCD-related deaths occur in these countries [3, 6].

Clinical practice guidelines (CPGs) influence the clinical practice. Several societies identify relevant clinical fields, formulate clinical questions, review the evidences, and formulate recommendations that health professionals and patients should follow [7]. Several CPGs have been developed worldwide, which increases concerns related to the quality of these guidelines. Indeed, studies have shown that many CPGs have only poor to moderate quality [8–11] and fail in terms of evaluating and making available resources for the implementation of recommendations on the part of healthcare services [12]. Different tools have been developed to enable the assessment of the quality of CPGs [13]. The Appraisal of Guidelines Research & Evaluation (AGREE) Instrument was developed by an international group. This assessment tool was first published in 2003 and updated in 2009 (AGREE II) [14]. The AGREE II has been widely used and provides a comprehensive, rapid, robust appraisal of CPGs [13–16].

Although previous studies have appraised the quality of CPGs [9, 17–20], there is a lack of a comprehensive systematic review and critical appraisal of CPGs for the treatment of NCDs. Therefore, the proposed study will systematically identify and appraise CPGs for the pharmacological treatment of most prevalent NCDs in primary care. The present study is an extension of our previous work, Molino et al. 2016 [8].

The primary objective is to assess the methodological rigor of the development and transparency of CPGs comprising pharmacological treatment for NCDs using the AGREE II instrument and identify CPGs of high quality. The secondary objective is to identify factors associated with the quality of these CPGs.

## Methods

### Protocol and registration

This study is registered in the PROSPERO database under protocol CRD42016043364 [21] and was prepared in compliance with the Preferred Reporting Items for Systematic Review and Meta-Analysis Protocols (PRISMA-P) [22], see Additional file 1.

### Literature search

A comprehensive literature search will be conducted in the following databases: MEDLINE (through PubMed), Embase and the Cochrane Library (through CENTRAL). The following databases specific to CPGs will also be searched: Australian Clinical Practice Guidelines (clinicalguidelines.gov.au), Brazilian Ministry of Health (saude.gov.br), Canadian Agency for Drugs and Technologies in Health (cadth.ca), Canadian Medical Association (cma.ca), Chilean Ministry of Health (bibliotecaminsal.cl/guias-clinicas-auge/), Colombian Ministry of Health and Social Protection (http://gpc.minsalud.gov.co/gpc/SitePages/default_gpc.aspx), Guidelines International Network (g-i-n.net), Institute for Clinical Systems Improvement (icsi.org), National Guideline Clearinghouse (guidelines.gov), Portal GuíaSalud (guiasalud.es), Scottish Intercollegiate Guidelines Network (sign.ac.uk), and the National Institute for Health and Care Excellence (nice.org.uk/). The sample search is shown in Additional file 2. The literature search involved the period from January 1, 2011, to December 31, 2016. The search was first conducted on May 24, 2016, and updated on January 22, 2017.

### Eligibility criteria

CPGs for the treatment of the following NCDs will be selected: asthma, atrial fibrillation, benign prostatic hyperplasia, chronic obstructive pulmonary disease, congestive heart failure, coronary artery disease and/or stable angina, dementia, depression, type 2 diabetes mellitus, gastroesophageal reflux disease, hypercholesterolemia, hypertension, osteoarthritis and osteoporosis [4, 5, 23–26]. A study will be considered a CPG when it comprises recommendations of pharmacological treatment for the treatment of the NCDs listed.

CPGs will be included if the pharmacological recommendations target adults or older adults; are written in English, Portuguese, or Spanish; and were published from January 1, 2011, to December 31, 2016. CPGs in which pharmacological recommendations are only one part will also be included. CPGs will be excluded for the following reasons: absence of pharmacological treatment recommendations; designed for local use, for example in a single health facility or single regional health service; designed for use with only hospitalized patients or patients in long-term care facilities; designed for a specific population within any of the listed NCDs, such as treatment of depression among individuals with cancer; the most recent version is unavailable; the full text is unavailable or only a summary of the recommendations is available; or the condition addressed is not on the pre-established list of NCDs. Documents that had only addressed the use of medications, for example, guidance about interventions to improve the appropriate use of/adherence to medications, will be also excluded.

### Clinical practice guideline selection and data extraction

**Fig. 1:**
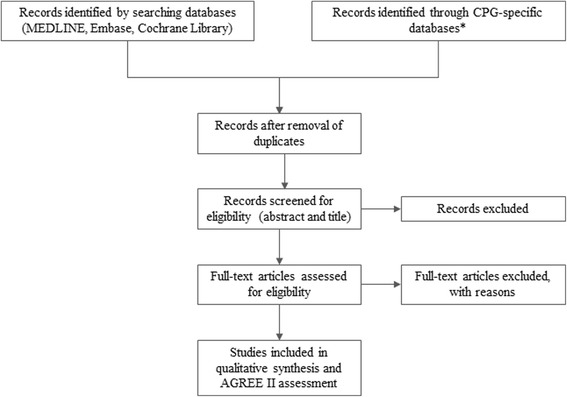
Flow diagram for selection of clinical practice guidelines according to PRISMA. *Specific databases for clinical practice guidelines (CPGs): Australian Clinical Practice Guidelines, Brazilian Ministry of Health, Canadian Agency for Drugs and Technologies in Health, Canadian Medical Association, Chilean Ministry of Health, Colombian Ministry of Health and Social Protection, Guidelines International Network, Institute for Clinical Systems Improvement, National Guideline Clearinghouse, Portal GuíaSalud, Scottish Intercollegiate Guidelines Network and the National Institute for Health and Care Excellence

Figure 1 shows the proposed CPG selection process. Potentially relevant records from the MEDLINE, Embase, and Cochrane Library databases will be retrieved and added to the Mendeley desktop® program. Duplicated studies will be excluded using the same software. The titles and abstracts will be independently screened by two reviewers, who will then conduct a full-text screening based on the eligibility criteria. Discrepancies will be resolved by a third reviewer.

Records from the CPG-specific databases will be added to an Excel® worksheet. One reviewer will conduct a full-text screening for duplicated records from the MEDLINE, Embase, and Cochrane Library databases. Two reviewers will then include records by consensus. Discrepancies that cannot be resolved through discussion will be referred to a third reviewer.

A hand search will also be performed in the publisher websites to identify and include the latest version and every document related to the selected CPGs, such as supplementary documents, summaries of the recommendations, documents aimed at patient education, and a previous version when mentioned in the CPG. For instance, the original publication of the CPG from the National Guideline Clearinghouse will be included and both publications will be assessed as one CPG. Moreover, CPGs considered withdrawn from the specific guidelines database will be included when such CPGs are still valid in the original publisher website.

Data extraction of the selected studies will be conducted independently by two reviewers using a standard form in Google Forms®. To perform the data extraction process, the reviewers will log onto their Google account, which will make it possible to track each reviewer response. Extracted data will be downloaded as an Excel® worksheet and the reviewers will check for agreement. Discrepancies will be resolved by consensus. If no consensus is reached, a third reviewer will make the decision.

The following data will be extracted: type of NCD, number of authors, year of publication or update, defined time to update, publisher and type of publisher (government, medical society, or university), type of guideline (formulated, adapted, updated, or review), country, funding, aim, target population/healthcare professional, scope (diagnostic, prevention, screening, pharmacological, or non-pharmacological treatment), type of study method (systematic review, consensus, overview), methods of formulating recommendation (consensus, not mentioned, other), and methods of grading evidence (GRADE [27], Oxford [28], not mentioned, other).

### Quality assessment

The quality of each CPG will be assessed using the Appraisal of Guidelines Research and Evaluation (AGREE) Instrument, version II (AGREE II) [14]. AGREE II was chosen as it allows a comprehensive appraisal of CPGs and is a widely accepted tool for the evaluation of the rigor of the development and transparency of CPGs [13–16]. The AGREE II that has been translated and validated in Portuguese will be used [29]. This tool comprises 23 items organized into six domains: (1) scope and purpose, (2) stakeholder involvement, (3) rigor of development, (4) clarity of presentation, (5) applicability, and (6) editorial independence. Each item is scored on a 7-point scale, for which 1 indicates a very poor report of the concept evaluated and 7 is attributed when all criteria and considerations are met [30].

Three appraisers will perform the quality assessment using the AGREE II instrument. Another reviewer will check the AGREE II results. Differences among scores of each item equal to or greater than 2 will be considered discrepant and the appraisers will decide the final score by consensus. If no consensus is reached, a fourth appraiser will decide the score.

**Fig. 2:**
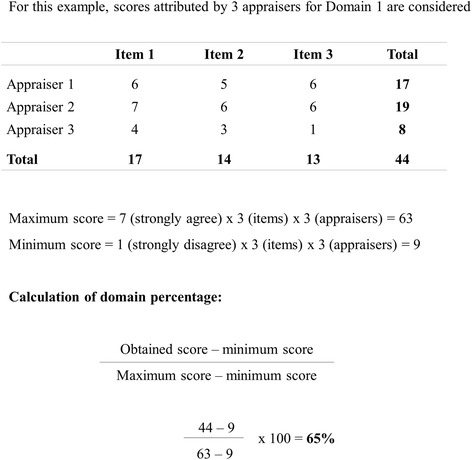
Example of AGREE II percentage calculation considering Domain 1 and 3 appraisers. This figure was adapted from the AGREE II User’s Manual [30]

The quality score of each CPG will be calculated per domain, as described in the AGREE II User’s Manual [30]. In summary, the six domains are independent and scores should therefore be calculated as the sum of the individual items in each domain, and then the total should be scaled as a percentage of the maximum possible score for the domain (Fig. 2). AGREE II appraisals will be conducted using the My AGREE PLUS platform, available at http://www.agreetrust.org/resource-centre/agree-plus/.

In addition to the six domains, the AGREE II instrument provides two overall assessments in which the appraisers score a CPG using the same 7-point scale and state whether they would recommend its use. As these assessments constitute a subjective analysis [9, 10], both overall assessments will not be considered for the purposes of statistical analysis.

### AGREE II training

The appraisers will be trained to use the AGREE II instrument in the following steps:

1. Study the AGREE II User’s Manual [30], a paper on the AGREE II validation in Brazil [29] and a paper offering an assessment of the Brazilian Ministry of Health guidelines [31]
2. Register with the My AGREE PLUS platform available at http://www.agreetrust.org/resource-centre/agree-plus/ and fill out the AGREE II Online Training Tool available at http://www.agreetrust.org/resource-centre/agree-ii-training-tools/
3. Appraise three CPGs of different levels of quality: two from the Brazilian Ministry of Health and one from the National Institute for Health and Care Excellence (NICE); discuss the results with another, previously trained appraiser
4. Appraise two additional CPGs: one from an American medical society and one from the National Guideline Clearinghouse (NGC); discuss the results with another, previously trained appraiser

### Strategy for data synthesis

In accordance with the Preferred Reporting Items for Systematic Reviews and Meta-Analyses (PRISMA) statement, the study selection process will be presented in a flow diagram (Fig. 1) describing the total of number of records found per database, total number of duplicates, records excluded based on the title and abstract, records excluded following the full-text screening with the rationale described, and total number of CPGs included. Each excluded study and the rationale for the exclusion will be provided as a supplementary document. The AGREE II scores will also be made available as a supplementary document.

The summary of the extracted data and AGREE scores will be presented in a descriptive table. Descriptive statistics will be calculated for all domains (mean, median, interquartile range). The data will be tested for normality and proper inferential tests will be conducted to analyze the magnitude and direction of associations between CPG quality and the extracted variables (year of publication, country, type of guideline, publisher, method of development, formulation of recommendation and grading evidence, applicability to older adults, and applicability to individuals with multimorbidity). Graphs will be plotted as needed. Two-sided *p* values less than 0.05 will be considered statistically significant. The statistical analysis will be conducted using the STATA 13® software.

## Discussion

As a result of the proposed study, we expect to identify high quality CPGs for NCDs using the AGREE II instrument and associated factors of high-quality CPGs. Moreover, future studies may also develop and disseminate a matrix of pharmacological recommendations for each NCD listed based on the high-quality CPGs identified. Finally, we believe the results of this study will be of great interest to other health institutions, CPG developers, and policy makers worldwide, helping them to select and adapt high-quality CPGs.

The research findings will be submitted for publication in high-impact, peer-reviewed scientific journals and will also be disseminated at international conferences. The PRISMA statement will be followed to report the study.

### Strengths and limitations

This review has several strengths. First, an extensive literature search will be conducted in 15 databases and all studies comprising relevant outcomes, recommendations, and the trade-off between the benefits and harms of interventions addressing NCDs will be included. Second, CPGs written in any of the three languages (English, Portuguese, and Spanish) will be included. Third, CPGs for prevalent NCDs will be evaluated. Fourth, two reviewers will perform the data extraction independently, which will comprise a variety of CPG characteristics. Fifth, the CPG appraisers will be extensively trained in the use of the AGREE II instrument. Sixth, three appraisers will assess CPGs using the AGREE II instrument. Seventh, a high degree of rigor will be used to verify discrepancies in the scores of AGREE II among the appraisers.

The limitations of this review might be the non-inclusion of CPGs without pharmacological treatment, in which had been written in other languages and published in databases other than those considered. Besides our comprehensive literature search, the results of our study may be influenced by publication bias. The quality of CPGs might be overestimated as we will search on bibliographic and specific guideline databases only. Poor-quality CPG may not be included in those databases. However, since studies showed that the quality of CPG may have a wide variation [9, 10], we considered that the publication bias might be minimal. Moreover, our results might be underestimated as CPGs from countries that have been developing CPG systematically, such as Germany, might not be included in our study due to the language restrictions.

## Additional files

Additional file 1: Preferred reporting items for systematic review and meta-analysis protocols 2015 statement (PRISMA-P checklist): required components to report in a systematic review protocol. (PDF 343 kb)

Additional file 2: Sample search: sample search for the database MEDLINE (through PubMed). (PDF 298 kb)
