# Supplementary material for: Methodological quality and transparency of clinical practice guidelines for the pharmacological treatment of non-communicable diseases using the AGREE II instrument: a systematic review protocol
Source: Syst Rev. 2017 Nov 2;6:220. doi: 10.1186/s13643-017-0621-5 (PMC5667495; doi:10.1186/s13643-017-0621-5)
Supplement: Supplementary file 2 — Sample search: sample search for the database MEDLINE (through PubMed). (PDF 298 kb) [file 13643_2017_621_MOESM2_ESM.pdf]

Sample search conducted on MEDLINE (Pubmed).

1	(((("Guideline" [Publication Type] OR "Guidelines as Topic"[Mesh] OR "Practice Guideline" [Publication Type] OR "Health Planning Guidelines"[Mesh]) OR "Clinical Protocols"[Mesh])) OR ("Consensus Development Conference, NIH" [Publication Type] OR "Consensus Development Conference" [Publication Type] OR "Consensus"[Mesh]))) OR "Standard of Care"[Mesh])) "Guideline" [Publication Type] OR "Guidelines as Topic"[Mesh] OR "Practice Guideline" [Publication Type] OR "Health Planning Guidelines"[Mesh]) OR "Clinical Protocols"[Mesh])) OR ("Consensus Development Conference, NIH" [Publication Type] OR "Consensus Development Conference" [Publication Type] OR "Consensus"[Mesh]))) OR "Standard of Care"[Mesh]))))
2	((("Hypertension"[Mesh] OR Blood Pressure, High OR Blood Pressures, High OR High Blood Pressure OR High Blood Pressures) OR ("Atrial Fibrillation"[Mesh] OR Atrial Fibrillations OR Fibrillation, Atrial OR Fibrillations, Atrial OR Familial Atrial Fibrillation OR Auricular Fibrillation OR Auricular Fibrillations OR Fibrillation, Auricular OR Fibrillations, Auricular) OR ("Heart Failure"[Mesh] OR Cardiac Failure OR Heart Decompensation OR Decompensation, Heart OR Heart Failure, Right-Sided OR Heart Failure, Right Sided OR Right-Sided Heart Failure OR Right Sided Heart Failure OR Myocardial Failure OR Congestive Heart Failure OR Heart Failure, Congestive OR Heart Failure, Left-Sided OR Heart Failure, Left Sided OR Left-Sided Heart Failure OR Left Sided Heart Failure) OR ("Angina Pectoris"[Mesh] OR "Angina, Stable"[Mesh] OR Stenocardia OR Stenocardias OR Angor Pectoris OR Anginas, Stable OR Stable Angina OR Stable Anginas OR Chronic Stable Angina OR Angina, Chronic Stable OR Anginas, Chronic Stable OR Chronic Stable Anginas OR Stable Angina, Chronic OR Stable Anginas, Chronic OR Angina Pectoris, Stable OR Angina Pectori, Stable OR Pectori, Stable Angina OR Pectoris, Stable Angina OR Stable Angina Pectori OR Stable Angina Pectoris) OR ("Coronary Disease"[Mesh] OR Coronary Diseases OR Disease, Coronary OR Diseases, Coronary OR Coronary Heart Disease OR Coronary Heart Diseases OR Disease, Coronary Heart OR Diseases, Coronary Heart OR Heart Disease, Coronary OR Heart Diseases, Coronary) OR ("Hypercholesterolemia"[Mesh] OR Hypercholesterolemias OR Elevated Cholesterol OR Hypercholesteremia OR Hypercholesteremias OR “Hypertriglyceridemia”[Mesh] OR Hypertriglyceridemias OR "Dyslipidemias"[Mesh] OR Dyslipidemia OR Dyslipoproteinemias OR Dyslipoproteinemia) OR ("Atherosclerosis"[Mesh] AND "Coronary Artery Disease"[Mesh] OR Artery Disease, Coronary OR Artery Diseases, Coronary OR Coronary Artery Diseases OR Disease, Coronary Artery OR Diseases, Coronary Artery OR Coronary Arteriosclerosis OR Arterioscleroses, Coronary OR Coronary Arteriosderoses OR Atherosderosis, Coronary OR Atheroscleroses, Coronary OR Coronary Atheroscleroses OR Coronary Atherosclerosis OR Arteriosclerosis, Coronary OR Atheroscleroses OR Atherogenesis))
3	("Diabetes Mellitus"[Mesh] OR “Diabetes Mellitus, Type 2"[Mesh] OR NIDDM OR Maturity-Onset Diabetes OR Diabetes Mellitus, Noninsulin-Dependent OR Diabetes Mellitus, Adult-Onset OR Adult-Onset Diabetes Mellitus OR Diabetes Mellitus, Adult Onset OR Diabetes Mellitus, Ketosis-Resistant OR Diabetes Mellitus, Ketosis Resistant OR Ketosis-Resistant Diabetes Mellitus OR Diabetes Mellitus, Maturity-Onset OR Diabetes Mellitus, Maturity Onset OR Diabetes Mellitus, Non Insulin Dependent OR Diabetes Mellitus, Non-Insulin-Dependent OR Non-Insulin-Dependent Diabetes Mellitus OR Diabetes Mellitus, Noninsulin Dependent OR Diabetes Mellitus, Slow-Onset OR Diabetes Mellitus, Slow Onset OR Slow-Onset Diabetes Mellitus OR Diabetes Mellitus, Stable OR Stable Diabetes Mellitus OR Diabetes Mellitus, Type II OR Maturity-Onset Diabetes Mellitus OR Maturity Onset Diabetes Mellitus OR MODY OR Type 2 Diabetes Mellitus OR Noninsulin-Dependent Diabetes Mellitus)
4	("Asthma"[Mesh] OR Asthmas OR Bronchial Asthma OR Asthma, Bronchial)
5	("Pulmonary Disease, Chronic Obstructive"[Mesh OR COPD OR Chronic Obstructive Pulmonary Disease OR COAD OR Chronic Obstructive Airway Disease OR Chronic Obstructive Lung Disease OR Airflow Obstruction, Chronic OR Airflow Obstructions, Chronic OR Chronic Airflow Obstructions OR Chronic Airflow Obstruction)
6	("Gastroesophageal Reflux"[Mesh OR Gastric Acid Reflux OR Acid Reflux, Gastric OR Reflux, Gastric Acid OR Gastric Acid Reflux Disease OR Gastro-Esophageal Reflux OR Gastro Esophageal Reflux OR Reflux, Gastro-Esophageal OR Gastroesophageal Reflux Disease OR GERD OR Reflux, Gastroesophageal OR Esophageal Reflux OR Gastro-oesophageal Reflux OR Gastro oesophageal Reflux OR Reflux, Gastro-oesophageal)

7	("Prostatic Hyperplasia"[Mesh] OR Hyperplasia, Prostatic OR Prostatic Hypertrophy OR Adenoma, Prostatic OR Adenomas, Prostatic OR Prostatic Adenomas OR Prostatic Adenoma OR Benign Prostatic Hyperplasia OR Prostatic Hyperplasia, Benign OR Prostatic Hypertrophy, Benign OR Benign Prostatic Hypertrophy OR Hypertrophy, Benign Prostatic)
8	("Osteoporosis"[Mesh] OR "Osteoporosis, Postmenopausal"[Mesh] OR Bone Loss, Postmenopausal OR Bone Losses, Postmenopausal OR Postmenopausal Bone Losses OR Postmenopausal Osteoporosis OR Osteoporoses, Postmenopausal OR Postmenopausal Osteoporoses OR Perimenopausal Bone Loss OR Postmenopausal Bone Loss OR Bone Loss, Perimenopausal OR Bone Losses, Perimenopausal OR Perimenopausal Bone Losses OR Osteoporosis, Post-Menopausal OR Osteoporoses, Post-Menopausal OR Osteoporosis, Post Menopausal OR Post-Menopausal Osteoporoses OR Post-Menopausal Osteoporosis OR Osteoporoses OR Osteoporosis, Post-Traumatic OR Osteoporosis, Post Traumatic OR Post-Traumatic Osteoporoses OR Post-Traumatic Osteoporosis OR Osteoporosis, Senile OR Osteoporoses, Senile OR Senile Osteoporoses OR Senile Osteoporosis OR Osteoporosis, Involutional OR Osteoporosis, Age-Related OR Osteoporosis, Age Related OR Bone Loss, Age-Related OR Age-Related Bone Loss OR Age-Related Bone Losses OR Bone Loss, Age Related OR Bone Losses, Age-Related OR Age-Related Osteoporosis OR Age Related Osteoporosis OR Age-Related Osteoporoses OR Osteoporoses, Age-Related)
9	("Dementia"[Mesh] AND "Alzheimer Disease"[Mesh] OR Dementias OR Amentia OR Amentias OR Senile Paranoid Dementia OR Dementias, Senile Paranoid OR Paranoid Dementia, Senile OR Paranoid Dementias, Senile OR Senile Paranoid Dementias OR Familial Dementia OR Dementia, Familial OR Dementias, Familial OR Familial Dementias OR Disease, Alzheimer OR Alzheimer Sclerosis OR Sclerosis, Alzheimer OR Alzheimer Syndrome OR Syndrome, Alzheimer OR Alzheimer Dementia (AD) OR Dementia, Alzheimer (AD) OR Alzheimer-Type Dementia (ATD) OR Alzheimer Type Dementia (ATD) OR Dementia, Alzheimer-Type (ATD) OR Primary Senile Degenerative Dementia OR Dementia, Senile OR Senile Dementia OR Dementia, Alzheimer Type OR Alzheimer Type Dementia OR Senile Dementia, Alzheimer Type OR Alzheimer Type Senile Dementia OR Dementia, Primary Senile Degenerative OR Alzheimer's Disease OR Disease, Alzheimer's OR Acute Confusional Senile Dementia OR Senile Dementia, Acute Confusional OR Dementia, Presenile OR Presenile Dementia OR Alzheimer Disease, Late Onset OR Late Onset Alzheimer Disease OR Alzheimer's Disease, Focal Onset OR Focal Onset Alzheimer's Disease OR Familial Alzheimer Disease (FAD) OR Alzheimer Disease, Early Onset OR Early Onset Alzheimer Disease OR Presenile Alzheimer Dementia)
10	("Depressive Disorder"[Mesh] AND "Depressive Disorder, Major"[Mesh] OR Depressive Disorders OR Disorder, Depressive OR Disorders, Depressive OR Neurosis, Depressive OR Depressive Neuroses OR Depressive Neurosis OR Neuroses, Depressive OR Depression, Endogenous OR Depressions, Endogenous OR Endogenous Depression OR Endogenous Depressions OR Depressive Syndrome OR Depressive Syndromes OR Syndrome, Depressive OR Syndromes, Depressive OR Depression, Neurotic OR Depressions, Neurotic OR Neurotic Depression OR Neurotic Depressions OR Melancholia OR Melancholias OR Unipolar Depression OR Depression, Unipolar OR Depressions, Unipolar OR Unipolar Depressions)
11	("Osteoarthritis"[Mesh] OR Osteoarthritides OR Osteoarthrosis OR Osteoarthroses OR Arthritis, Degenerative OR Arthritides, Degenerative OR Degenerative Arthritides OR Degenerative Arthritis OR Osteoarthrosis Deformans)

12	("Atrial Fibrillation"[Mesh] OR Atrial Fibrillations OR Fibrillation, Atrial OR Fibrillations, Atrial OR Familial Atrial Fibrillation OR Auricular Fibrillation OR Auricular Fibrillations OR Fibrillation, Auricular OR Fibrillations, Auricular) OR ("Heart Failure"[Mesh] OR Cardiac Failure OR Heart Decompensation OR Decompensation, Heart OR Heart Failure, Right-Sided OR Heart Failure, Right Sided OR Right-Sided Heart Failure OR Right Sided Heart Failure OR Myocardial Failure OR Congestive Heart Failure OR Heart Failure, Congestive OR Heart Failure, Left-Sided OR Heart Failure, Left Sided OR Left-Sided Heart Failure OR Left Sided Heart Failure) OR ("Angina Pectoris"[Mesh] OR "Angina, Stable"[Mesh] OR Stenocardia OR Stenocardias OR Angor Pectoris OR Anginas, Stable OR Stable Angina OR Stable Anginas OR Chronic Stable Angina OR Angina, Chronic Stable OR Anginas, Chronic Stable OR Chronic Stable Anginas OR Stable Angina, Chronic OR Stable Anginas, Chronic OR Angina Pectoris, Stable OR Angina Pectori, Stable OR Pectori, Stable Angina OR Pectoris, Stable Angina OR Stable Angina Pectori OR Stable Angina Pectoris) OR ("Coronary Disease"[Mesh] OR Coronary Diseases OR Disease, Coronary OR Diseases, Coronary OR Coronary Heart Disease OR Coronary Heart Diseases OR Disease, Coronary Heart OR Diseases, Coronary Heart OR Heart Disease, Coronary OR Heart Diseases, Coronary) OR ("Hypercholesterolemia"[Mesh] OR Hypercholesterolemias OR Elevated Cholesterol OR Hypercholesteremia OR Hypercholesteremias OR "Hypertriglyceridemia"[Mesh] OR Hypertriglyceridemias OR "Dyslipidemias"[Mesh] OR Dyslipidemia OR Dyslipoproteinemias OR Dyslipoproteinemia) OR ("Atherosclerosis"[Mesh] AND "Coronary Artery Disease"[Mesh] OR Artery Disease, Coronary OR Artery Diseases, Coronary OR Coronary Artery Diseases OR Disease, Coronary Artery OR Diseases, Coronary Artery OR Coronary Arteriosclerosis OR Arterioscleroses, Coronary OR Coronary Arterioscleroses OR Atherosclerosis, Coronary OR Atheroscleroses, Coronary OR Coronary Atheroscleroses OR Coronary Atherosclerosis OR Arteriosclerosis, Coronary OR Atheroscleroses OR Atherogenesis)
13	#1 AND #2
14	#1 AND #3
15	#1 AND #4
16	#1 AND #5
17	#1 AND #6
18	#1 AND #7
19	#1 AND #8
20	#1 AND #9
21	#1 AND #10
22	#1 AND #11
23	#1 AND #12
24	#13 OR #14 OR #15 OR #16 OR #17 OR #18 OR #19 OR #20 OR #21 OR #22 OR #23
25	#24 Filters: Publication date from 2011/01/01 to 2016/12/31
